# Isoquinoline alkaloids isolated from *Glaucium corniculatum* var. *corniculatum* and *Glaucium grandiflorum* subsp. *refractum* var. *torquatum* with bioactivity studies

**DOI:** 10.1080/13880209.2023.2218881

**Published:** 2023-06-19

**Authors:** Tuba Kusman Saygi, Nur Tan, Gülbahar Özge Alim Toraman, Caglayan Unsal Gurer, Osman Tugay, Gulacti Topcu

**Affiliations:** a Department of Pharmacognosy, Faculty of Pharmacy, Bezmialem Vakif University, Istanbul, Turkey; b Department of Pharmacognosy, Institute of Health Sciences, Istanbul University, Istanbul, Turkey; c Department of Medicinal and Aromatic Plants, Hamidiye Vocational School of Health Services, University of Health Sciences, Istanbul, Turkey; d Center for Research and Practice in Drug Development from Natural Sources, Istanbul University, Istanbul, Turkey; e Department of Pharmaceutical Botany, Faculty of Pharmacy, Selcuk University, Konya, Turkey; f Drug Application and Research Center (DARC), Bezmialem Vakif University, Istanbul, Turkey

**Keywords:** Glaucium, glauciumoline, glaucine, aporphine, protopine, structure elucidation

## Abstract

**Context:**

The genus *Glaucium* Mill., one of the important Papaveraceae family plants, is rich in isoquinoline alkaloids and distributed worldwide.

**Objective:**

Isolation and identification of bioactive alkaloids from *Glaucium grandiflorum* Boiss. & Huet. subsp. *refractum* (Nabelek) Mory var. *torquatum* (Cullen) Mory and *G. corniculatum* (L.) Rudolph var. *corniculatum* (Aslan 2012)*,* and investigation of their antioxidant and anticholinesterase activities.

**Materials and methods:**

The aerial parts of each plant were dried, powdered, and percolated with methanol, then each extract was fractionated between 50% aqueous acetic acid and petroleum. Their aqueous acidic layer was adjusted to pH 7–8 with NH_4_OH and extracted with chloroform, the extract was subjected to CC separation and isolation. Structures of the isolated alkaloids were elucidated by 1D and 2D-NMR and mass spectral analyses. The alkaloid extracts and their pure alkaloids were tested for anti-cholinesterase (AChE and BuChE) and antioxidant (ABTS, CUPRAC, β-carotene linoleic acid tests) activities *in vitro*.

**Results:**

Methanol extracts of *Glaucium grandiflorum* subsp. *refractum* var. *torquatum* and *G. corniculatum* var. *corniculatum* afforded a novel compound glauciumoline and seven known isoquinoline alkaloids three of which have an aporphine-type and the other five have a protopine-type skeleton. Among them, *trans*-protopinium (**7**) and *cis*-protopinium (**8**) were isolated from a *Glaucium* species for the first time. Tertiary amine extracts (TAEs) of both plants showed very strong acetylcholinesterase inhibitory activity. The TAE of the plants also showed strong antioxidant activity while the isolated alkaloids showed no meaningful activity in the anticholinesterase and antioxidant tests.

**Discussion and conclusions:**

*Glaucium* species are considered promising therapeutic agents in the treatment of Alzheimer’s disease.

## Introduction

*Glaucium* Mill. (Papaveraceae) is one of the most diverse genera, distributed worldwide. The genus includes about 28 species (Kadereit 1993; Mingli and Grey-Wilson 2008; Aykurt et al. 2017; Akaberi et al. 2021) native to southern Europe and Mediterranean region, and central and south-western Asia. Most of the *Glaucium* species mainly grown in Iran (17 species) (Mory 1979), and Turkey (12 taxa) (Aslan 2012; Yıldırımlı 2012; Yıldız et al. 2015; Aykurt et al. 2017).

In the Flora of Turkey, the genus *Glaucium* is represented by 12 taxa, 7 of which are endemic. *Glaucium grandiflorum* Boiss.& Huet. has two varieties, var. *grandiflorum* Boiss.& Huet. and var. *torquatum* Cullen, the latter is an endemic species. *Glaucium corniculatum* (L.) Rud. is one of the biodiverse species with three subspecies; subsp. *phoeniceum* (Crantz) Holmboe, subsp. *refractum* (Nábelek) Cullen, subsp. *tricolor* (Besser) Holmboe and seven varieties; var. *caricum* (Stapf) Kuntze, var. *corniculatum* (L.) Rudolph, var. *flavum* (Crantz) Kuntze, var. *fulvum* (Sm.) Kuntze, var. *grandiflorum* (Boiss. & A.Huet) Kuntze, var. *leiocarpum* (Boiss.) Kuntze, var. *pilosum* Kuntze (Güner et al. 2012).

The genus is well known for its various pharmacological and biological activities including antimicrobial, anti-inflammatory, antitussive, antioxidant, hypoglycemic, bronchodilator and cytotoxic activities (Arafa et al. 2016). *Glaucium* species have been used as laxative, sedative, hypnotic, antidiabetic purposes and in the treatment of dermatitis (Zargari 1990; Shafiee and Morteza-Semnani 1998). In another study, the antibacterial activity of the methanol and chloroform extracts of the aerial parts of three *Glaucium* species; *G. grandiflorum*, *G. oxylobum* and *G. paucilobum* has been tested using the disk diffusion method and exhibited concentration-dependent activity against a series bacteria (Morteza-Semnani et al. 2005).

In folk medicine, fruits of *Glaucium grandiflorum* have been used for purification of blood and in the treatment of ophthalmic diseases (Baytop 1994). *G. flavum* Crantz., is one of the most studied *Glaucium* species which shows antitussive, antioxidant (Spasova et al. 2008), hypoglycemic (Cabo et al. 1988) and hypotensive effects (Orallo et al. 1995).

Turkish *Glaucium* species were first investigated by the Gozler group for isolation and structure elucidation of their alkaloids (Coşar et al. 1981; Gozler 1982). However, their biological activities were mostly investigated by other researchers later on (Sari 2001; Ozsoy et al. 2018).

Aerial parts of the *Glaucium* species are very rich in alkaloids, particularly aporphine-type isoquinoline alkaloids. Aporphinoids were also isolated from roots of Algerian *Glaucium flavum* which showed anti-tumour effects against human cancer cells (Bournine et al. 2013).

Glaucine has been used commonly as an antitussive and antihypertensive agent for a long time (Dimant and Bardashevskaia 1974). Glaucine was also widely known for antitussive, bronchodilatory and anti-inflammatory properties (Chiu et al. 2012), and its drugs found in eastearn European market, especially in Bulgaria as cough suppressant (Meyer et al. 2013) Glaucine act also as weak dopamine D_1_ and D_2_ receptor antagonist (Meyer et al. 2013). The antitussive effect of glaucine is comparable to codeine without signs of opiate withdrawal after long intake (Zhang et al. 2007).

In one study, glaucine was found to be a significant cytotoxic agent against cervical cancer HeLa cells (Hoet et al. 2004). In another study, a series of 18 aporphinoids, including glaucine, have been investigated *in vitro* against human poliovirus and found to be significantly active (Chen et al. 2013). Aporphine alkaloids can further inhibit calcium ion channels. They caused relaxation of the thoracic aorta of rats by suppressing the Ca^2+^ influx through both voltage- and receptor-operated calcium channels (Ivorra et al. 1993; Eltze et al. 2002).

**Figure 1.**
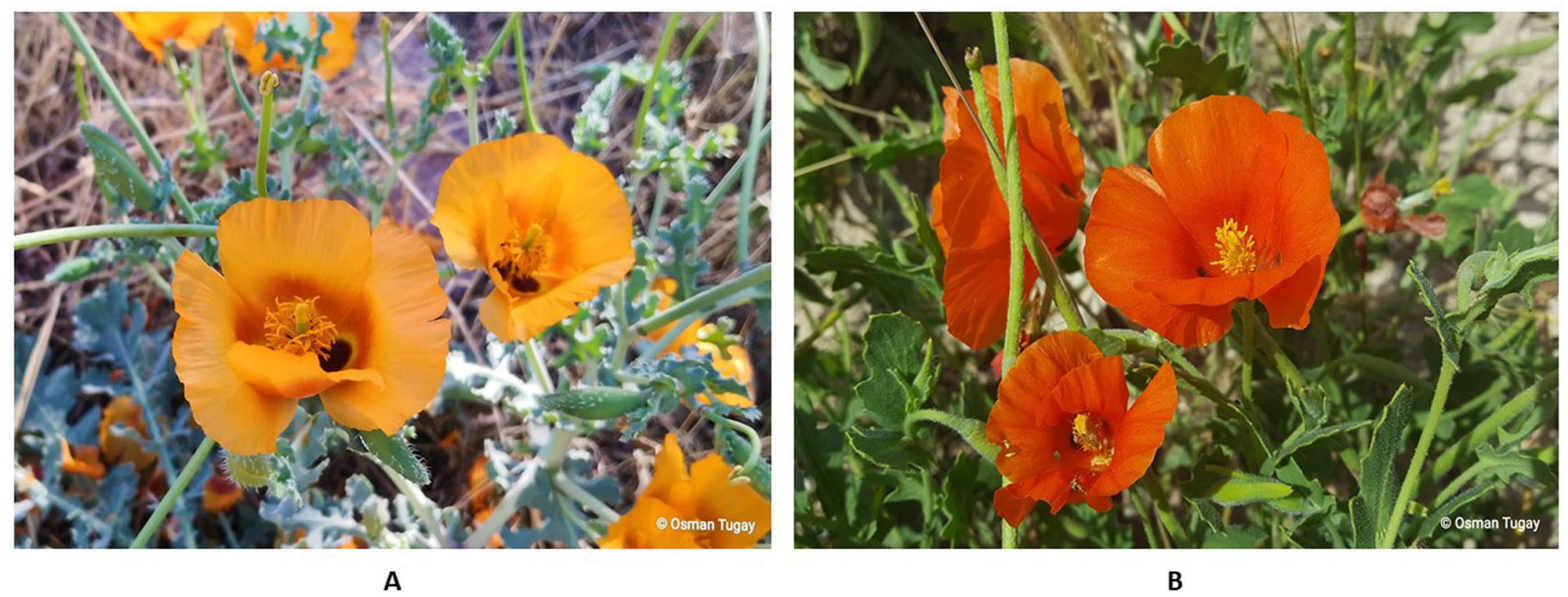
Images of some *Glaucium* species. A*: Glaucium grandiflorum* subsp. *refractum* var. *torquatum* (Aslan 2012)*;* B: *Glaucium corniculatum* var. *corniculatum.*

Glaucine was found to be the major alkaloid of *Glaucium* species, especially in the species *G. flavum* (Arafa et al. 2016). However, in this study, glaucine was not obtained from the extracts of the endemic species *G. grandiflorum* subsp. *refractum* var. *torquatum* (Aslan 2012)*,* while *G. corniculatum* var. *corniculatum* (Figure 1) afforded glaucine with a low yield (0.94% = 45 mg/4.75 g tertiary amine extract [TAE]) (Kusman 2018). A earlier study, carried out by Gozler (1982) on the alkaloid extracts of former species, afforded glaucine at high yield (70%), which was collected from Ankara, the capital city of Turkey. This result should be attributed to the collection of the plant from a different location and a different season as well as other factors (Bogdanov et al. 2015).

In this study, the extracts and isolated alkaloids from the two *Glaucium* species were investigated phytochemically with their bioactivity. In the literature, only a few anticholinesterase and antioxidant activity studies have been performed with alkaloid extracts of *Glaucium* species rather than their isolated alkaloids (Akaberi et al. 2021). However, the selected two *Glaucium* species have not been investigated for their anticholinesterase and antioxidant activities heretofore. Therefore, investigation of both species, particularly endemic species *G. grandiflorum* subsp. *refractum* var*.* *torquatum* (Aslan 2012), for the mentioned activities, would be worthwhile.

## Materials and methods

### General experimental procedure

^1^H- and ^13^C-NMR spectra were recorded on a Varian ID-6508 at 600 MHz for proton and 150 MHz for carbon with tetramethylsilane (TMS) as an internal standard. Some NMR spectra, particularly 2D NMR, were run with a Bruker Avance 500 MHz in the Drug Application and Research Center (DARC), Bezmialem Vakif University, Istanbul, Turkey. Chemical shifts values are given in ppm (δ scale), coupling constants(*J*) in Hz.

Optical rotations were measured on Rudolph Research Analytical, Autopol V Plus with AutofillTM Automatic Polarimeter. Measurements were taken at a temperature of 25 °C and a wavelength of 589 nm. Compounds concentrations were set to 0.267 g/100 mL.

Spots and bands were detected with a UV Camag spectrometer (254 and 366 nm). Mass spectral measurements were taken in Zivak^®^ Tandem Gold Triple quadrupole (Istanbul, Turkey) mass spectrometer. HRMS spectra were run on Thermo ORBITRAP Q-EXACTIVE in Drug Application and Research Center (DARC). Column chromatography was conducted with silica gel 60 (0.063–0.200 mm) (Merck No: 1.07734). Thin-layer chromatography (TLC) was performed on silica gel 60 F_254_ plates (Merck No: 1.05554).

Methanol, chloroform, cyclohexane, diethylamine were purchased from Merck.

### Plant material

*Glaucium grandiflorum* Boiss. & Huet. subsp. *refractum* var. *torquatum* (Aslan 2012) Cullen was collected at the flowering stage from Van (Eastern of Turkey, 38° 5.72″ N, 43° 2.75″ E) in May, 2016 and identified by Prof. Dr. Şükran Kültür (the plant specimen is preserved in the ISTE Herb.; ISTE 110.338), and *G. corniculatum* (L.) Rud. var. *corniculatum* was collected at the flowering stage from Konya (Center of Turkey, 38° 01.125″ N, 32° 30.033″ E) in June, 2016, and identified by Prof. Dr. Osman Tugay (the plant specimen is preserved in the KNYA Herb.; KNYA 30.113).

### Extraction, fractionation and isolation

Air-dried and powdered aerial parts of *Glaucium grandiflorum* subsp. *refractum* var. *torquatum* (Aslan 2012) (1008.9 g) were percolated with methanol and the dried methanol extracts was obtained as 26.00 g (yield 2.6%). It was then fractionated between 50% aqueous acetic acid and petroleum ether. The aqueous acidic layer was adjusted to pH 7–8 with NH_4_OH (conc.) and extracted with CHCI_3_ (×6), collected chloroform layers were dried over Na_2_SO_4_, and the solvents were evaporated. The crude chloroform extract (1.2 g) was chromatographed on a column of silica gel. Dried chloroform extract elution was started with CHCl_3_ 100%, and continued addition of MeOH as gradient [CHCl_3_:MeOH (9:1, 8:2, 7:3, 5:5)] successively, and then finally MeOH (100%) to collect 39 fractions. The obtained eluates were further purified using preparative TLC on silica gel with cyclohexane:diethylamine (8:2) or chloroform:cyclohexane:diethylamine (7:3:1). All compounds were identified by ^1^H- and ^13^C NMR techniques and Mass spectrometric analyses.

Air-dried and powdered aerial parts of *G. corniculatum* var. *corniculatum* (534.50 g) were percolated with methanol and then evaporated. Dried methanol extract was obtained as 132.00 g (yield 25%). The same extraction procedure used for the *Glaucium corniculatum* var. *corniculatum* was also applied for this species. The crude chloroform extract (4.75 g) of *G. corniculatum* var. *corniculatum* was chromatographed on a silica gel column. The column elution was started with CHCl_3_, and gradients CHCl_3_:MeOH mixtures (9:1, 8:2, 7:3, 5:5), and then only MeOH solvent, successively, and 62 fractions were collected. The similar fractions were combined and were further purified using preparative TLC Si-gel plates which developed cyclohexane:diethylamine (8:2) or chloroform:cyclohexane:diethylamine (7:3:1) solvents system.

### Antioxidant activity

Antioxidant activities of the extracts and pure compounds were investigated for β*-*carotene-linoleic acid (Miller 1971), ABTS cation radical decolorization (Re et al. 1999) and cupric reducing antioxidant capacity (CUPRAC) (Apak et al. 2004) methods. The antioxidant activity of the extracts and compounds were compared with the standards of BHT, BHA and α*-*tocopherol.

#### β-Carotene lipid peroxidation inhibition method

Lipid peroxidation inhibition test method is also known as total antioxidant activity. This method is based on measuring the inhibition of conjugated diene hydroperoxides resulting from linoleic acid oxidation and the principle of lightening β*-*carotene (Miller 1971).

#### ABTS cation radical scavenging activity method

In this test assay, green/blue coloured ABTS radical is formed by oxidation of ABTS with persulphate. The method is based on the principle that the ABTS radical decolouration at 600–750 nm wavelength by reduction. The free radical scavenging activity of antioxidant substances is calculated as equivalent to Trolox and the results are given as the ‘TEAC value’ (Re et al. 1999).

#### Cupric-reducing antioxidant capacity method (CUPRAC)

According to this antioxidant method, neocuproin (2,9-dimethyl-1,10-phenanthroline) and Cu (II) are placed in the same well. The analysis was performed using Cu (I), which is formed by the reduction of Cu (II) by the antioxidant substance, giving a maximum absorbance at a wavelength of 450 nm by formation of complex with neocuproin, calculated as equivalent to Trolox, and the result is given as the ‘TEAC value’ (Apak et al. 2004).

### Anticholinesterase activity

Acetylcholinesterase (AChE) and butyrylcholinesterase (BChE) inhibitory activities of the extracts and isolated compounds were measured by slightly modifying the spectrophotometric method developed by Ellman et al. (1961). Galantamine was used as a reference compound.

### Statistical analysis

All bioactivity data were obtained in triplicate and the presented statistical data were analysed using Graphpad Prism version 9.00 (GraphPad Software, La Jolla CA).

## Results

**Figure 2.**
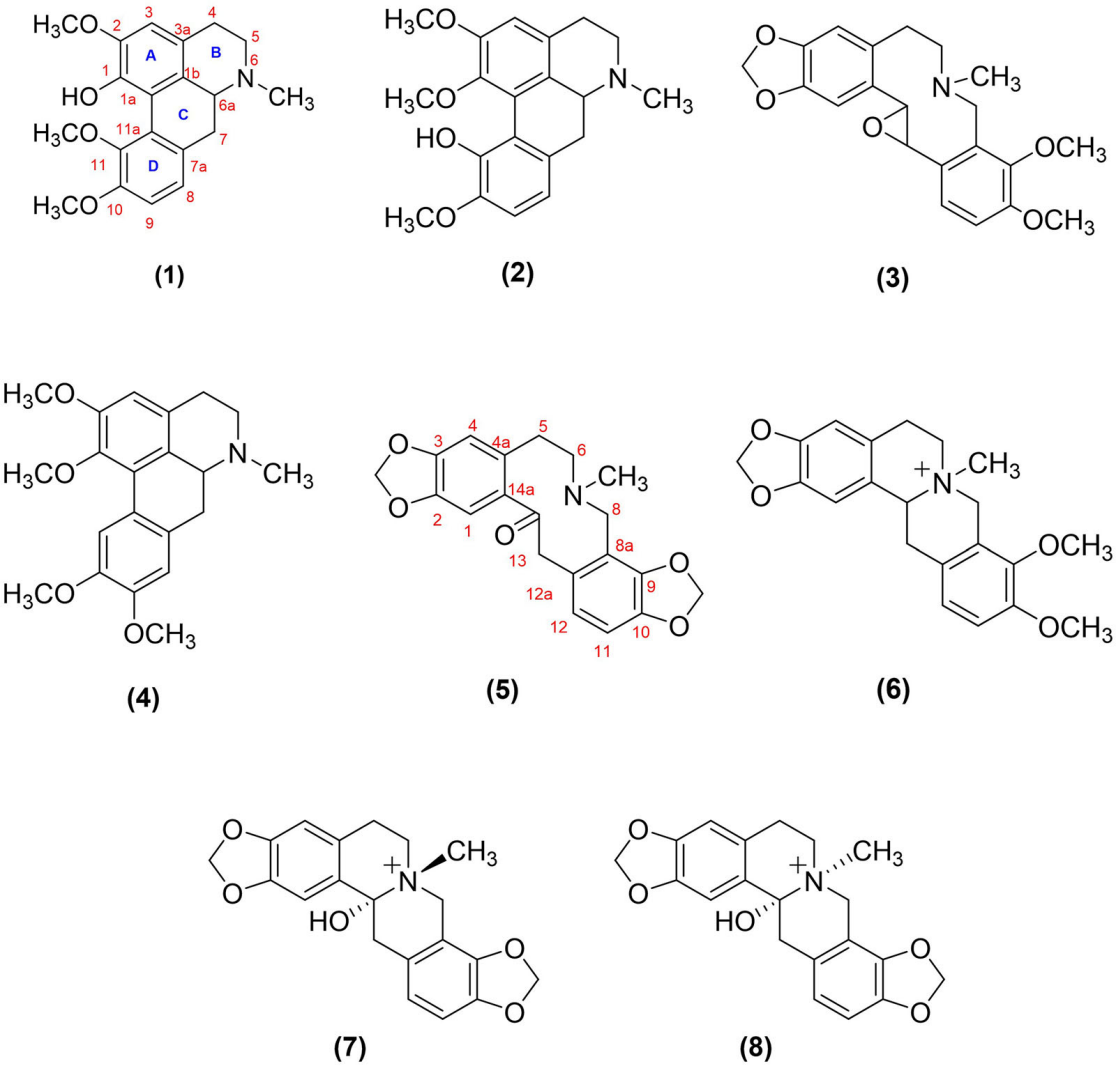
Structures of the compounds isolated from *G. grandiflorum* subsp. *refractum* var. *torquatum* (Aslan 2012)* and G. corniculatum* var. *corniculatum.*

In this study, two known aporphine alkaloids, corydine (**1**), and isocorydine (**2**), and two protopine-type alkaloids; one known as protopine (**5**), another one the new alkaloid glauciumoline (**3**), were isolated (Figure 2; Table 1) from the aerial parts of the *G. grandiflorum* subsp. *refractum* var. *torquatum* (Aslan 2012)*,* collected from Van, a city located in the Eastern-Anatolia. The other *Glaucium* species, *G. corniculatum* var. *corniculatum* (aerial parts), collected from Konya, afforded five alkaloids. These are a known aporhine alkaloid glaucine **(4)** and three known protopine type alkaloids; *N*-methyl canadine (**6**), *trans-*protopinium (**7**) and *cis-*protopinium (**8**), in addition to the new protopine-type alkaloid, glauciumoline **(3)** (Figure 2; Table 1).

**Table 1.** Amounts of the isolated alkaloids from *Glaucium* species.

*Glaucium* species	Alkaloids	Amounts of alkaloids (mg)
*Glaucium grandiflorum* Boiss. & Huet. subsp. *refractum* var. *torquatum* Cullen (Aslan 2012).	Corydine **(1)**	12
	Isocorydine **(2)**	8
	Glauciumoline **(3)**	10
	Protopine **(5)**	15
*Glaucium corniculatum* (L.) Rud. var. *corniculatum*	Glauciumoline **(3)**	16
	Glaucine **(4)**	45
	*N*-Methyl canadine **(6)**	30
	*trans-*Protopinium **(7)**	20
	*cis-*Protopinium **(8)**	27

Their structures were elucidated based on spectral analyses, particularly by 1D- and 2D ^1^H- and ^13^C-NMR experiments and mass spectroscopic measurements. Although three known aporphine alkaloids (glaucine, corydine and isocorydine) and two known protopine alkaloids (**5** and **6**) were previously isolated from different *Glaucium* species, compound **3** was isolated from nature for the first time, and isolation of compounds **7** and **8** was isolated for the first time from a *Glaucium* species, in this study.

Compound **3** was isolated as a yellow amorphous solid, and its possible alkaloid structure was proven by the observation of a positive test result after spraying Dragendorff’s reagent.

The ^1^H NMR spectrum of compound **3** (Table 2) exhibited a characteristic two proton singlet at δ 5.95, and the appearance of a carbon chemical shift (δ 102.92) in the ^13^C NMR spectrum attributed to the presence of a dioxo methylene group in the skeleton. It should be positioned either on ring A or ring D. In the ^1^H NMR spectrum, four aromatic proton signals resonated at δ 6.98, 6.96, 6.94 and 6.68. Among them, two proton signals as singlets at δ 6.98 and 6.68 which could be attributed to H-1 and H-4, respectively. Their correlated carbon signals (C-1 and C-4) observed at δ 107.30 and 109.03, respectively, based on HSQC experiments. The resonance of a proton singlet at δ 6.68 (H-4) showed two-bond and three-bond away correlations with a carbon signal at 134.07 ppm corresponding to C-4a and C-14a in the HMBC spectrum which verified the location of dioxo methylene group on ring A (between C-2 and C-3). The ^13^C NMR spectrum (Table 2) by an APT experiment showed the presence of 21 carbon signals consisting of a methylene dioxo moiety, two methoxy groups, three methylene pairs, 2 aliphatic and 4 aromatic methines, eight aromatic quaternary carbons, and a *N*-methyl signal in the molecule.

**Table 2.** ^1^H and ^13^C NMR data of protopine alkaloids glauciumoline (3), protopine (5), *N*-methyl canadine (6), *trans-*protopinium (7) and *cis-*protopinium (8).

Carbon	3 (CD_3_OD)		5 (CDCl_3_)		6 (CD_3_OD)		7 (CD_3_OD)		8 (CD_3_OD)	
	δH	δC	δH	δC	δH	δC	δH	δC	δH	δC
**1**	6.98 s, 1H	107.30	6.93 s, 1H	110.52	6.88 s, 1H	113.14	7.11 s, 1H	107.56	7.05 s, 1H	107.11
**2**	–	146.47	–	148.84	–	145.84	–	148.92	–	150.71
**3**	–	151.29	–	150.82	–	150.31	–	150.51	–	149.29
**4**	6.68 s, 1H	109.03	6.63 s, 1H	112.78	7.16 s, 1H	110.78	6.76 s, 1H	110.07	6.69 s, 1H	109.73
**4a**	–	134.07	–	135.20	–	125.01	–	n.d.	–	124.08
**5**	3.13 m, 1H 2.94 m, 1H	29.34	n.d.	32.01	3.38 m, 2H	25.15	3.20 bd, 2H	27.00	3.09 m, 1H 3.32 bd, 1H	25.20
**6**	3.20 m, 1H 2.77 m, 1H	55.09	3.90 m, 2H	54.21	3.62 m, 1H 3.68 m, 1H	55.72	3.45 bd, 2H	55.99	4.48 d (*J =* 16.0 Hz), 2H	56.37
**8**	4.08 m, 1H 3.98 m, 1H	51.79	3.91 m, 2H	59.97	4.62 s, 1H 3.87 s, 1H	56.19	3.62 m, 2H	55.47	3.80 m, 1H 3.53 m, 1H	55.39
**8a**	–	n. d.	–	130.70	–	124.49	–	n.d.	–	126.05
**9**	–	151.29	–	148.73	–	148.05	–	148.00	–	148.10
**10**	–	146.39	–	148.67	–	152.07	–	146.14	–	145.77
**11**	6.96 d (*J =* 7.8 Hz), 1H	111.37	6.70 d (*J =* 7.9 Hz), 1H	109.77	6.87 d (*J =* 7.8 Hz), 1H	109.88	6.84 d (*J =* 8.0 Hz), 1H	109.53	6.78 d, (*J =* 8.2 Hz), 1H	110.01
**12**	7.04 d (*J =* 7.8 Hz), 1H	126.58	6.66 d (*J =* 7.9 Hz), 1H	127.28	6.84 d (*J =* 7.8 Hz), 1H	123.76	6.80 d (*J =* 8.0 Hz), 1H	124.21	6.73 d, (*J =* 7.9 Hz), 1H	123.68
**12a**	–	n.d.	–	130.80	–	124.49	–	n.d.	–	124.08
**13**	3.32 bs, 1H	48.92	3.63 bs, 2H	32.35	3.45 m, 1H 3.19 bd, 1H	31.95	3.88 bs, 2H	31.50	1.20, 2H	30.91
**14**	3.92 m, 1H	59.66	–	n.d.	3.97 m, 1H	55.72	–	n.d.	–	n.d.
**14a**	–	134.07	–	129.10	–	125.31	–	n.d.	–	110.50
**N-CH_3_**	2.23 s, 3H	39.65	2.13 s, 3H	44.50	3.05 s, 3H	43.55	2.88 s, 3H	43.29	2.96 s, 3H	43.70
**2,3-O-CH_2_-O**	5.95 s, 2H	101.26	5.95 s, 2H	103.68	6.03 s, 2H	103.51	6.01 s, 2H	103.34	5.91 s, 2H	103.38
**9,10-O-CH_2_-O**	–	–	5.93 s, 2H	103.93	–	–	5.99 s, 2H	103.26	5.94 s, 2H	103.58
**9-OCH_3_**	3.83 s, 3H	54.84	–	–	3.88 s, 3H	56.65	–	–	–	–
**10-OCH_3_**	3.78 s, 3H	59.66	–	–	3.86 s, 3H	56.94	–	–	–	–

n.d.: not detected.

**Figure 3.**
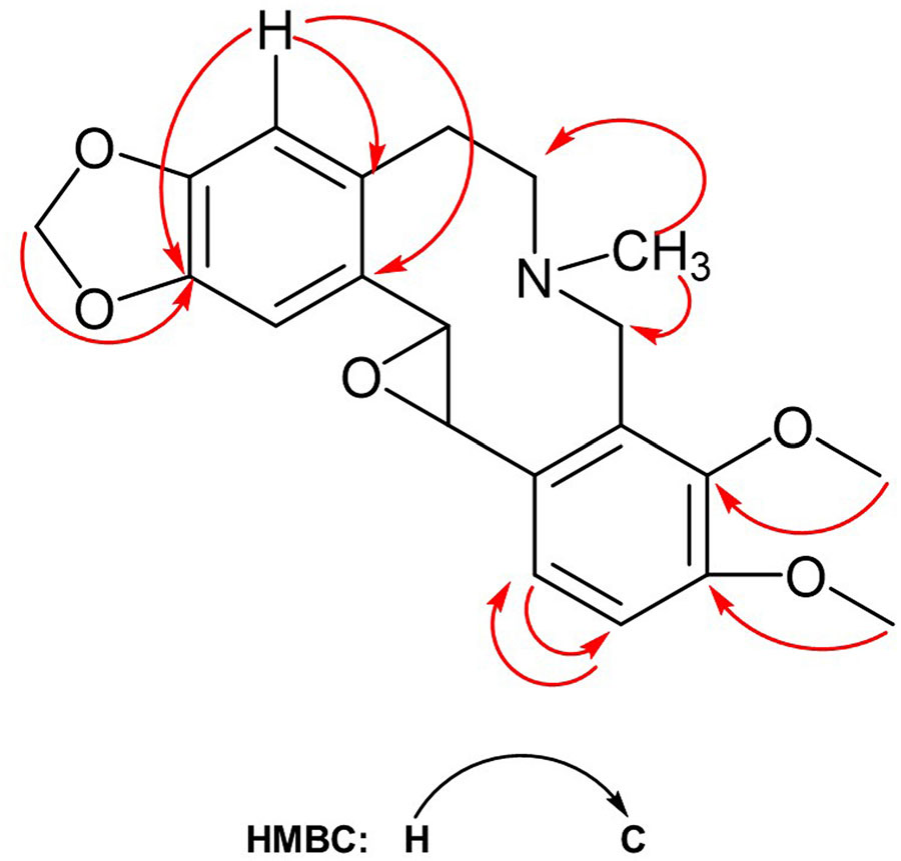
HMBC correlations of the glauciumoline (**3**).

The two distinct methoxy signals, resonated at δ 3.83 and 3.78, should be located at C-9 and C-10. This pattern is properly assigned by their carbon signals correlations in the HSQC and HMBC (Figure 3) experiments. The other two aromatic proton signals appeared as two doublets which have an AB spin system coupled to each other with a characteristic ortho *J* coupling value (7.8 Hz) which should be attached to C-9 and C-10 protons on ring D. The two methoxy protons and their corresponding carbons were determined based on HSQC experiments, and by HMBC experiments observing two-bond and three-bond away correlations (Figure 3) from H to C atoms.

The presence of an epoxy group in the structure of compound **3** was observed by two proton signals resonated at δ 3.32 brs (H-13) and 3.92 m (C-14) which coupled each other. Their vicinity followed by 1D and 2D NMR experiments. Their carbon signals resonated at δ 48.92 (C-13) and at δ 59.66 (C-14) which were correlated with their proton signals by the HSQC experiment. In the ^1^H NMR spectrum, three methylene pairs were observed; C-5 methylene pair with proton signals at δ 3.13 and 2.94 as multiplets corresponding to a C signal at δ 29.34; and for C-6 methylene pair with at δ 3.20 and 2.77 protons as multiplets corresponding to a C signal at δ 55.09; and for C-8 assignment with at δ 4.08 and 3.98 protons as multiplets corresponding to a C signal at δ 51.79 based on the ^13^C-NMR, HSQC, and HMBC experiments. *N*-Methyl protons resonated at δ 2.23 and C signal at δ 39.65 and showed three-bond away correlations with both C-6 and C-8 in the HMBC.

In fact, the structure of compound **3** resembled a protopine-type alkaloid based on above NMR data with a ten-member ring formed by B and C rings, which looks like allocryptopine (Kubala et al. 2011). But, instead of an oxo group, an epoxy group is present between C-13 and C-14, followed by two methine signals and their carbon signals mentioned above. The HRMS spectrum showed a peak at *m/z* 370.16330 [M + H]^+^ corresponding to the formula C_21_H_24_NO_5_ (theoretical mass: 370.16490) with 10.5 relative double bound (RDB) equivalent. Considering the complete assignment of protons and carbons through the aid of the 1D- and 2D NMR spectra and high-resolution mass spectral (HRMS) analysis results, the structure of the new compound **3** was elucidated, and the trivial name was given as glauciumoline.

Antioxidant activities of each extract and their alkaloids were investigated by three test (ABTS, CUPRAC and lipid peroxidation inhibition) methods.

**Figure 4.**
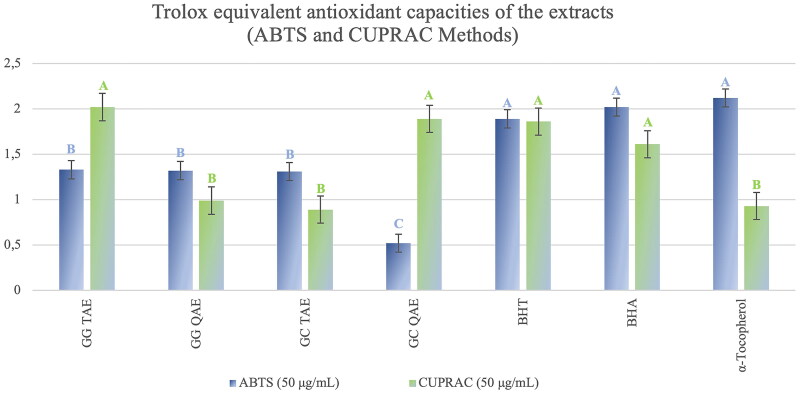
Trolox Equivalent Antioxidant Capacities of the extracts calculated with respect to the ABTS and CUPRAC methods. *G. grandiflorum* subsp. *refractum* var. *torquatum* (Aslan 2012) tertiary amine extract: GG TAE; *G. corniculatum* var. *corniculatum* tertiary amine extract: GC TAE; *G. grandiflorum* subsp. *refractum* var. *torquatum* (Aslan 2012) tertiary amine extract: GG TAE; *G. grandiflorum* subsp. *refractum* var. *torquatum* (Aslan 2012) quaternary amine extract: GG QAE. Test results for each extract shown with superscript capital letters (A–C) indicate significant differences (*p*<0.05) according to the Fisher test.

As antioxidant activity test assays, the extracts showed moderate activity in the ABTS method. In the CUPRAC antioxidant capacity test, *G. grandiflorum* subsp. *refractum* var. *torquatum* (Aslan 2012) extract showed strong activity while *G. corniculatum* var. *corniculatum* extract showed moderate activity at the same concentration (50 μg/mL) (Figure 4).

According to the ABTS activity results carried out at a concentration of 50 μg/mL, tertiary and quaternary amine extracts of *G. grandiflorum* subsp. *refractum* var. *torquatum* (Aslan 2012) showed strong antioxidant activity (1.33 ± 0.12 and 1.32 ± 0.24, respectively) compared to the standards (Figure 4) as well as the TAE of *G. corniculatum* var*. corniculatum* (1.31 ± 0.54), but quaternary amine extract of *G. corniculatum* var*. corniculatum* showed a moderate activity (Figure 4).

According to the CUPRAC activity test results, TAE of *G. grandiflorum* subsp. *refractum* var. *torquatum* (Aslan 2012) showed very strong antioxidant activity (2.02 ± 0.01 mol/g) at a concentration of 50 μg/mL, even higher than standard compounds (BHT, BHA, and α-tocopherol) as well as quaternary amine extract of *G. corniculatum* var. *corniculatum.* Therefore, the extracts showed high CUPRAC capacity except for *G. corniculatum* var. *corniculatum* TAE (Figure 4).

**Figure 5.**
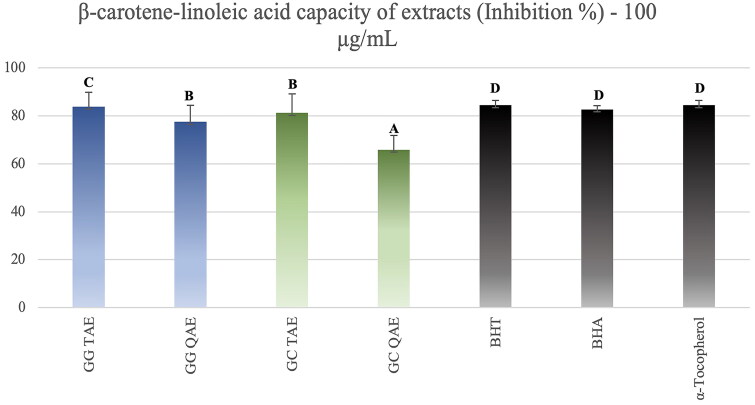
β*-*Carotene lipid peroxidation inhibition results of the *Glaucium* extracts. *G. grandiflorum* var.* torquatum* tertiary amine extract: GG TAE; *G. grandiflorum* subsp. *refractum*var. *torquatum* (Aslan 2012) quaternary amine extract: GG QAE; *G. corniculatum* var. *corniculatum* tertiary amine extract: GC TAE; *G*. *corniculatum* subsp. *refractum* var. *corniculatum* (Aslan 2012) quaternary amine extract: GC QAE; Test results for each extract shown with superscript capital letters (A–D) indicate significant differences (*p*<0.05) according to the Fisher test.

Considering β*-*carotene lipid peroxidation inhibition activity test results of the extracts obtained from *Glaucium* species, it was seen that TAEs of both plants showed high activity compared with the standards (Figure 5). Quaternary amine extracts also showed fairly high β*-*carotene-linoleic acid test results.

**Figure 6.**
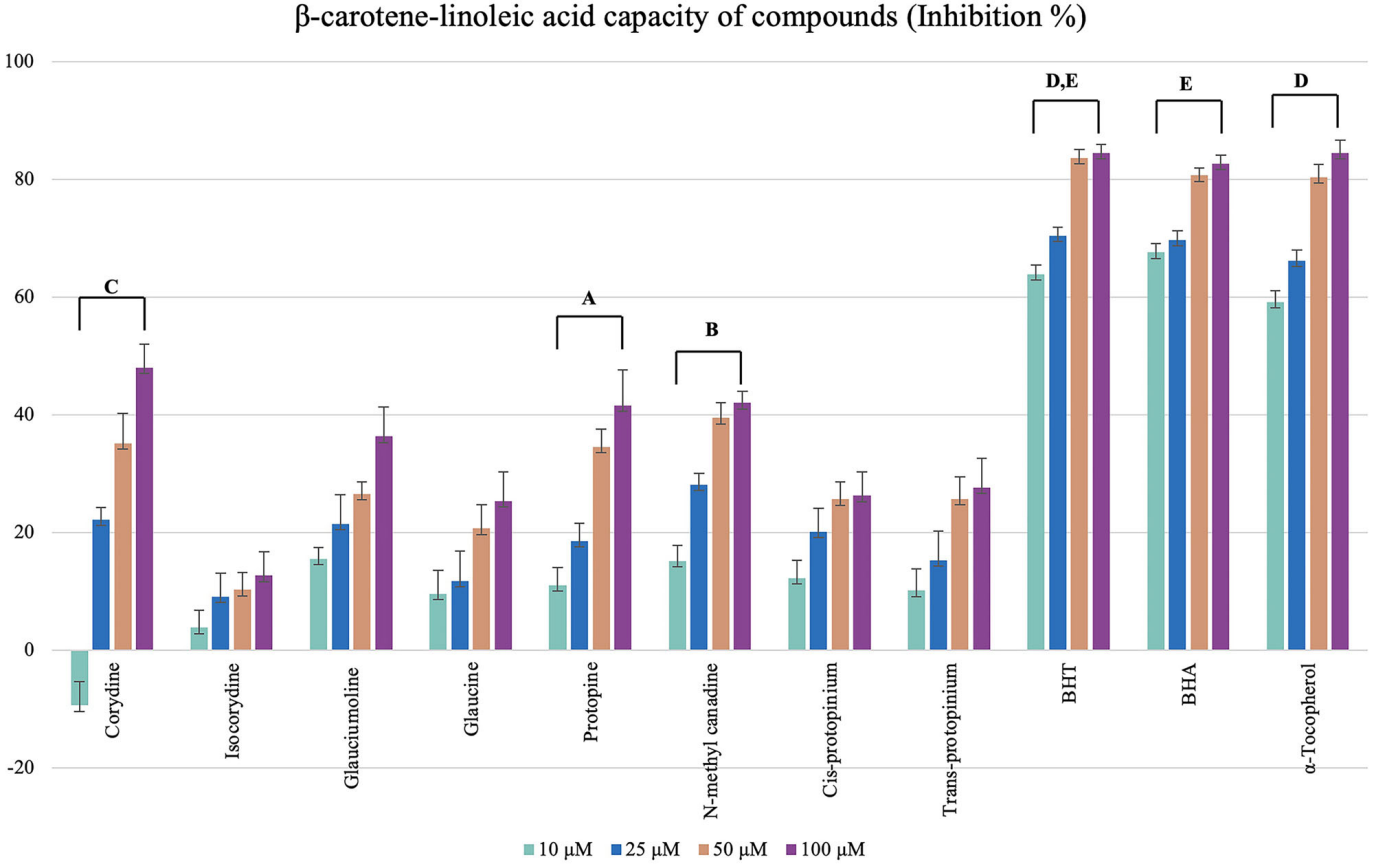
Lipid peroxidation inhibition results of the alkaloids from the *Glaucium* species. BHT, BHA, and α -Tocopherol were used as positive standards). Test results for each compound shown with superscript capital letters (A–E) indicate significant differences (*p*<0.001) according to the Fisher test.

However, none of the alkaloids showed activity in the ABTS and CUPRAC methods which were tested at 50 μg/mL. The isolated alkaloids tested for lipid peroxidation inhibitory activity by the β*-*carotene-linoleic acid test assay, corydine **(1)** showed relatively good activity with 48.02% inhibition at 100 μM, and *N*-methyl canadine **(6)** and protopine **(5)** followed (Figure 6).

**Figure 7.**
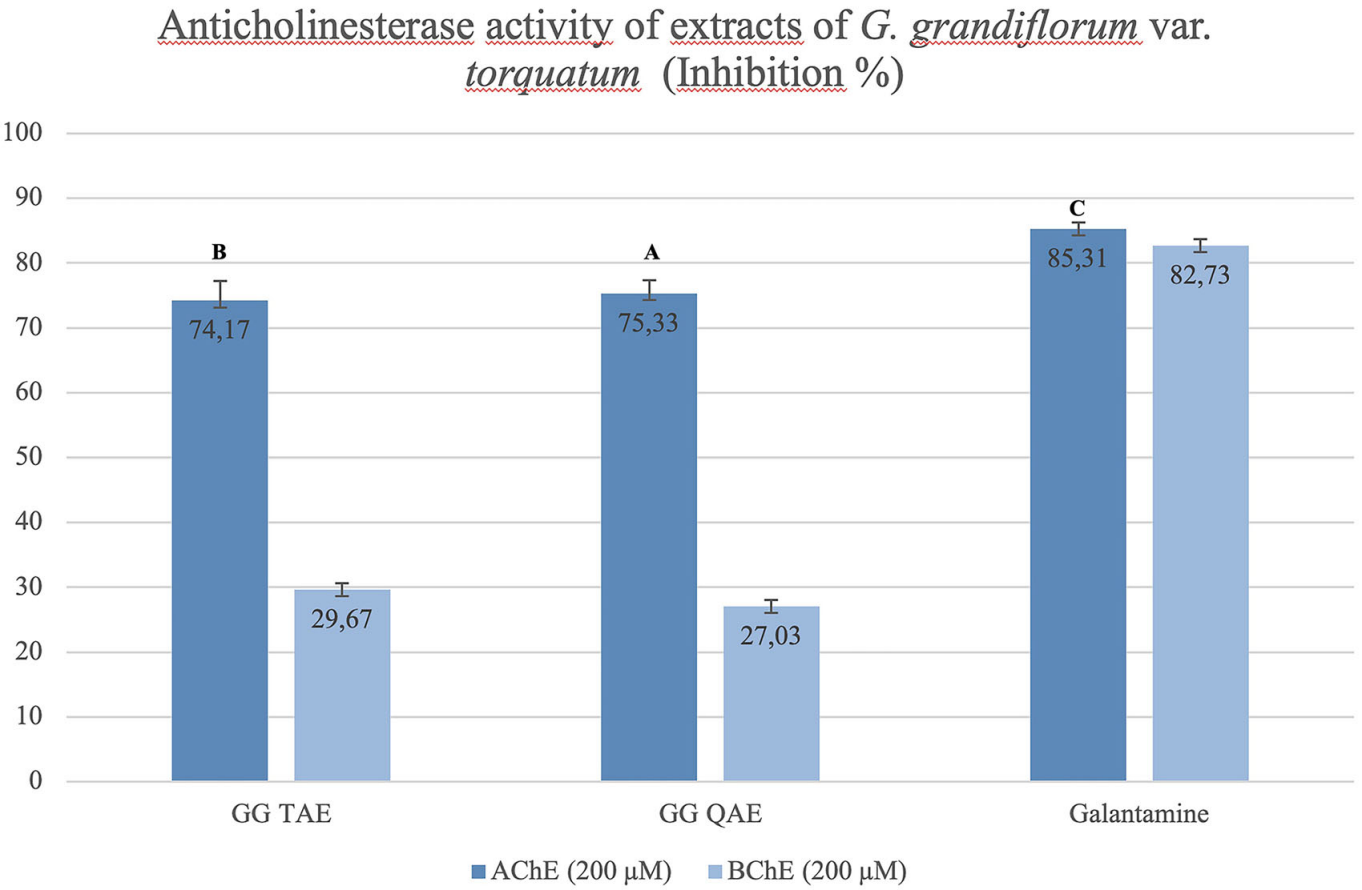
Anticholinesterase activity results of the *G. grandiflorum* subsp. *refractum* var. *torquatum* (Aslan 2012) extracts. GG TAE: Tertiary amine extract; GG QAE: Quaternary amine extract. Test results for each extract shown with superscript capital letters (A–E) indicate significant differences (*p*<0.05) according to the Fisher test.

The anticholinesterase activity of tertiary and quaternary amine extracts of *G. grandiflorum* subsp. *refractum* var. *torquatum* (Aslan 2012) were tested, and both extracts showed high inhibition (74.17% and 75.33% at 200 μM, respectively) on AChE enzyme compared with the galantamine. In contrast, the tertiary and quaternary amine extracts showed weak inhibition against the BChE enzyme (29.67% and 27.03% at 200 μM, respectively) (Figure 7).

**Figure 8.**
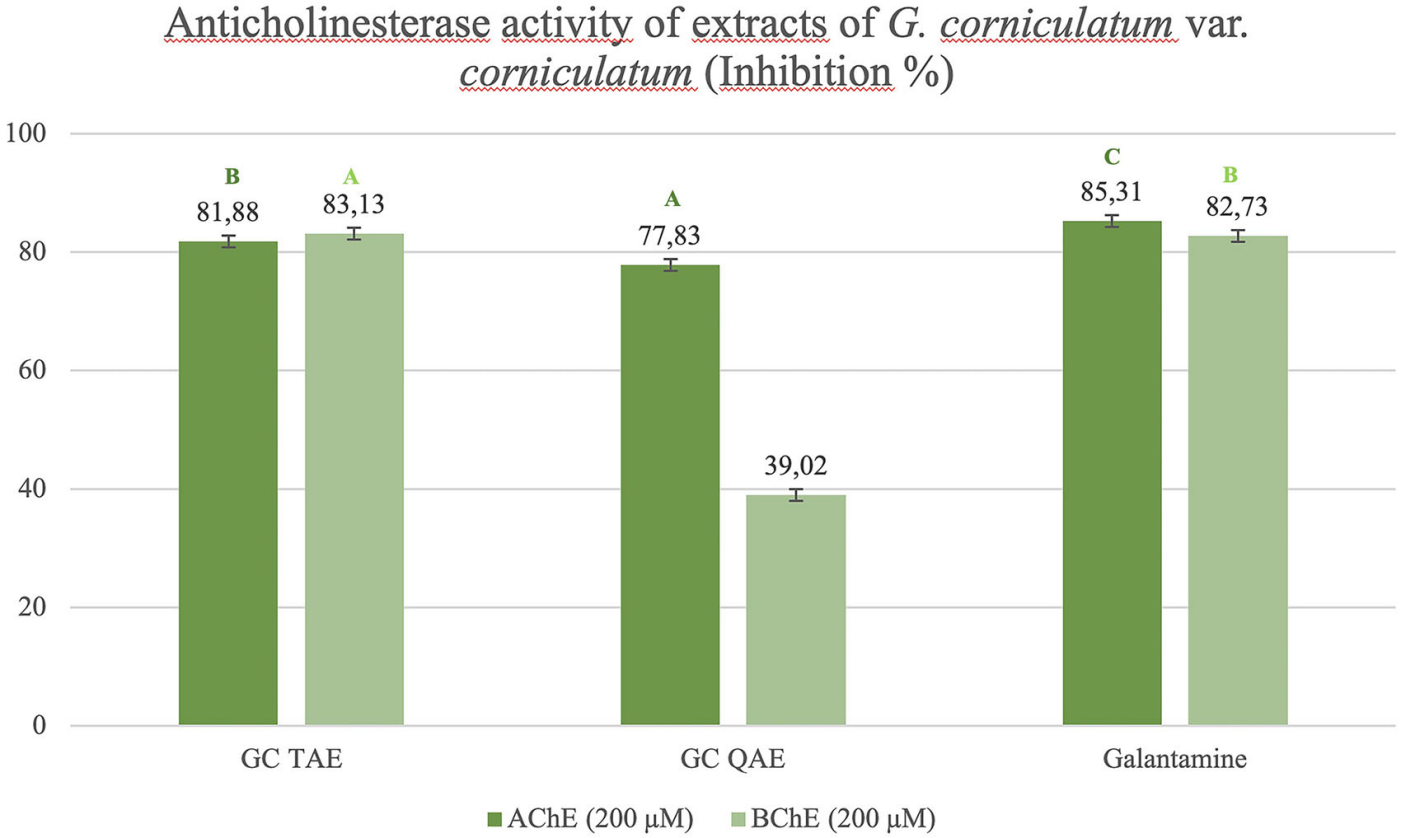
Anticholinesterase inhibition activity results of the *G. corniculatum* var*. corniculatum* extracts. GC TAE: Tertiary amine extract; GC QAE: Quaternary amine extract. Test results for each extract shown with superscript capital letters (A–E) indicate significant differences (*p*<0.01) according to the Fisher test.

**Figure 9.**
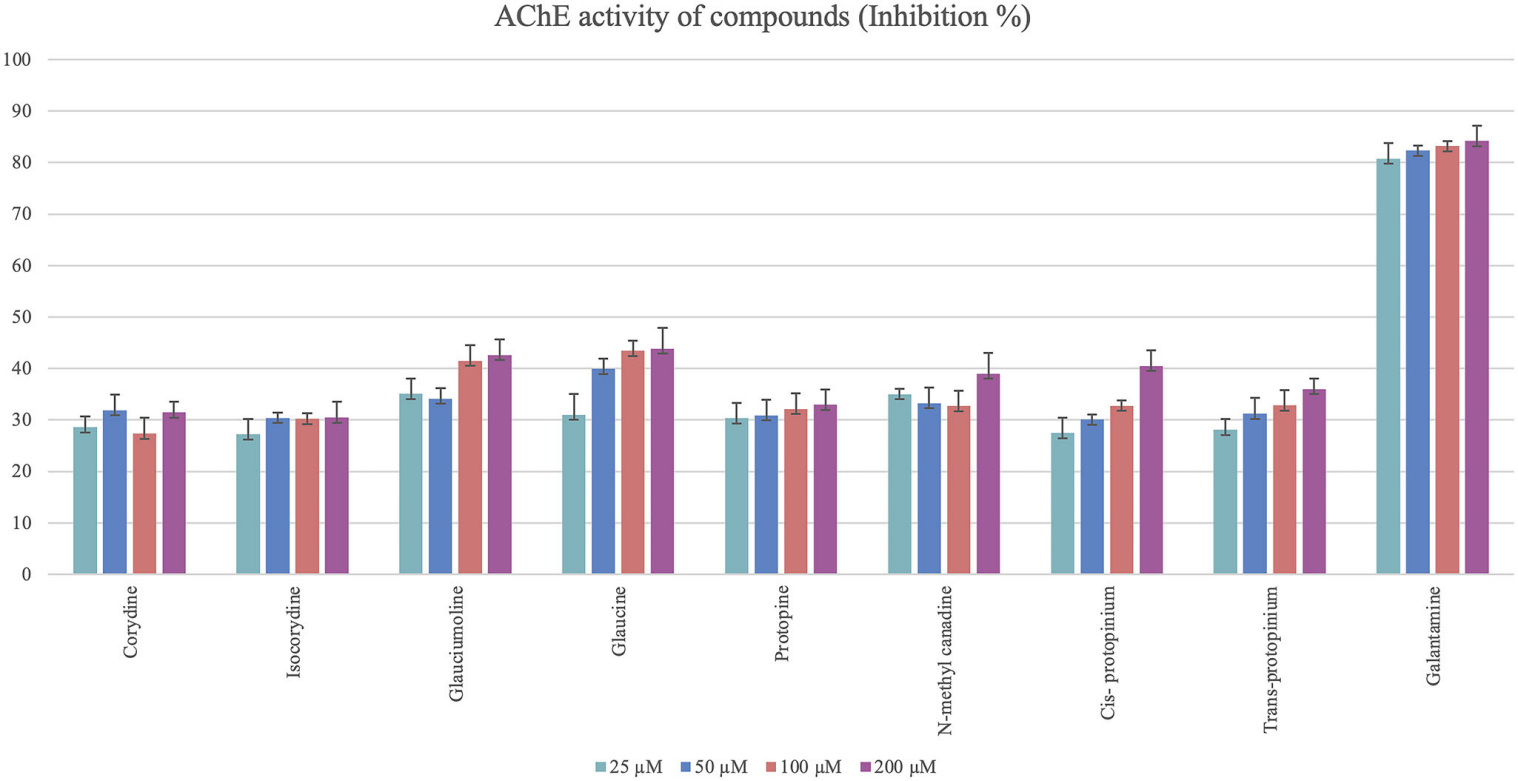
Acetylcholinesterase (AChE) inhibition activity results of isolated alkaloids from the *Glaucium* species. Statistical analysis was not performed because the test substances were not active.

The anticholinesterase activity test results of the *G. corniculatum* var. *corniculatum* extract were examined and the TAE showed high inhibition against AChE and BChE enzymes which is equivalent to that of galantamine (81.88% and 83.13% at 200 μM, respectively). Quaternary amine extract of *G. corniculatum* var. *corniculatum* also showed high inhibition on the AChE enzyme compared with the galantamine (77.83% inhibition at 200 μM) (Figure 8). However, the isolated alkaloids showed almost no anticholinesterase activity, except for *cis*-protopinium (Figure 9).

**Figure 10.**
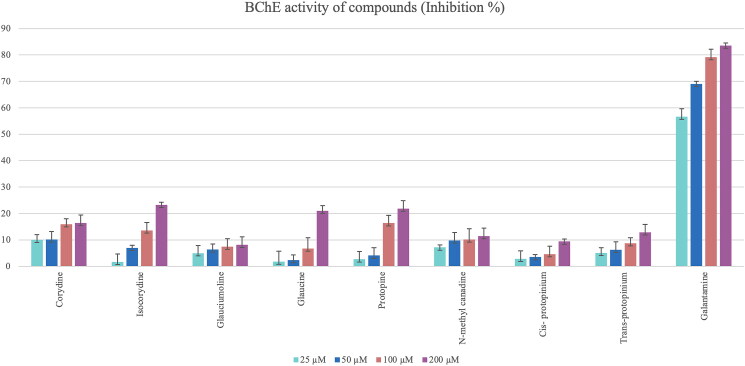
Butyrylcholinesterase (BChE) inhibitory activity results of the isolated alkaloids from the *Glaucium* species. Statistical analysis was not performed because the test substances were not active.

Glaucine and the new compound glauciumoline showed the highest AChE inhibitory activity among all tested alkaloids (Figure 9) while BChE inhibition activity results of the isolated alkaloids were found to be very low. Isocorydine, protopine and glaucine showed only weak inhibition on BChE enzyme at a concentration of 200 μM (Figure 10).

Compound ***1:*** ^1^H and ^13^C NMR data in Table 3. ESI-MS (–) *m/z* (%) = 341 [M]^+^ (calcd for C_20_H_23_NO_4_): 342 [M + H]^+^, 340 [M-H]^+^, 326 [M-CH_3_]^+^, 323 [M-H_2_O]^+^, 310 [M-OCH_3_]^+^, 298 [M-C_2_H_5_N]^+^, 279 [(M-OCH_3_)-OCH_3_]^+^.

**Table 3.** ^1^H and ^13^C NMR data of aporphine alkaloids corydine (**1**), isocorydine (**2**) and glaucine (**4**).

Carbon	1 (CDCl_3_)		2 (CDCl_3_)		4 (CD_3_OD)	
	δH	δC	δH	δC	δH	δC
**1**	–	151.70	–	146.57	–	146.07
**1a**	–	129.83	–	121.83	–	126.84
**1b**	–	129.85	–	127.00	–	128.15
**2**	–	140.78	–	154.86	–	154.08
**3**	6.70 s, 1H	111.29	6.71 s, 1H	113.80	6.64 s, 1H	112.14
**3a**	–	120.30	–	127.68	–	129.66
**4**	2.54 m, 2H	29.91	2.76 m, 2H	25.34	2.58 m, 1H 3.05 dd, 1H (*J =* 4.1; 13.5 Hz)	29.27
**5**	3.05 dd, (*J =* 12.9; 3.6 Hz), 2H	52.88	3.13 d, (*J =* 12.3 Hz), 2H	34.56	2.68 (*J =* 15.3; 3.0 Hz), 1H 3.09 dd (*J =* 4.7; 8.8 Hz), 1H	54.25
**6a**	3.24 m, 1H	62.60	3.30 m, 1H	58.75	3.12 m, 1H	56.51
**7**	2.72 bd, (*J =* 2.3; 16.4 Hz), 2H	35.80	2.76 m, 2H	27.52	2.45 t (*J =* 12.9 Hz), 1H 3.63 m, 1H	43.67
**7a**	–	126.20	–	127.37	–	130.35
**8**	6.82 d (*J =* 8.8 Hz), 1H	119.26	6.90 d, (*J =* 8.2 Hz), 1H	113.99	6.82 s, 1H	112.84
**9**	6.85 d (*J =* 8.2 Hz), 1H	111.36	7.12 d, *(J =* 8.2 Hz), 1H	127.69	–	150.12
**10**	–	144.26	–	154.86	–	149.25
**11**	–	15	–	146.57	7.89 s, 1H	113.63
**11a**	–	126.20	–	122.14	–	125.77
**N-CH_3_**	2.57 s, 3H	43.90	2.72 s, 3H	46.00	2.55 s, 3H	43.84
**1-OCH_3_**	–	–	3.91 s, 3H	58.82	3.78 s, 3H	56.60
**2-OCH_3_**	3.70 s, 3H	62.31	3.92 s, 3H	58.72	3.76 s, 3H	56.84
**9-OCH_3_**	–	–	–	–	3.75 s, 3H	60.67
**10-OCH_3_**	3.90 s, 3H	56.13	3.74 s, 3H	64.74	3.53 s, 3H	63.99
**11-OCH_3_**	3.91 s, 3H	56.39	–	–	–	–
**O-CH_2_-O**	–	–	–	–	–	–

n.d.: not detected.

Compound ***2:*** ^1^H and ^13^C NMR data in Table 3. ESI-MS (–) *m/z* (%) 341 [M]^+^ (calcd for C_20_H_23_NO_4_): 342 [M + H]^+^, 340 [M-H]^+^, 326 [M-CH_3_]^+^, 323 [M-H_2_O]^+^, 310 [M-OCH_3_]^+^, 298 [M-C_2_H_5_N]^+^, 283 [298-CH_3_]^+^.

Compound ***3:*** [α]^24,3^_D_**:** −2,25^0^. ^1^H and ^13^C NMR data are in Table 2. HRMS spectrometer (ORBITRAP Q-EXACTIVE), ESI (+) *m/z* 370.16330 [M + H]^+^ C_21_H_24_NO_5_ (RDB equiv. value: 10.5), (theoretical mass: 370.16490), 279.15790 (C_16_H_23_O_4_), 163.03813 (C_9_H_7_O_3_).

Compound ***4:*** ^1^H and ^13^C NMR data in Table 3. ESI-MS (-) *m/z* (%) = 355 [M]^+^ (calcd for C_21_H_25_NO_4_): 355 [M]^+^, 356 [M + H]^+^, 354 [M-H]^+^, 340 [M-CH_3_]^+^, 338 [M-H_2_O]^+^, 324 [M-OCH_3_]^+^, 297 [(M-OCH_3_)-CO]^+^.

Compound ***5:*** ^1^H and ^13^C NMR data in Table 2. ESI-MS (–) *m/z* (%) =353 [M]^+^ (calcd for C_20_H_19_NO_5_): 353 [M]^+,^ 354 [M + H]^+^, 323 [M-OCH_2_]+, 206, 148.

Compound ***6:*** ^1^H and ^13^C NMR data in Table 2. ESI-MS (–) *m/z* (%) = 354 [M]^+^ (calcd for C_21_H_24_NO_4_): 354 [M]^+^, 355 [M + H]^+^, 339 [M-CH_3_]^+^, 336 [M-H_2_O]^+^.

Compound ***7:*** ^1^H and ^13^C NMR data in Table 2. ESI-MS (–) *m/z* (%) = 355 [M]^+^ (calcd for C_20_H_21_NO_5_): 355 [M]^+^, 356 [M + H]^+^, 332, 320, 188.

Compound ***8:*** ^1^H and ^13^C NMR data in Table 2. ESI-MS (–) *m/z* (%) = 355 [M]^+^ (calcd for C_20_H_21_NO_5_): 355 [M]^+^, 356 [M + H]^+^, 332, 320, 188.

## Discussion

The aerial parts of *Glaucium* species contain a large amount of isoquinoline alkaloids, most of which have an aporphine ring or a protopine ring. Although *Glaucium* species were found to be rich in an aporphine alkaloid glaucine (Israilov et al. 1979; Daskalova et al. 1988), in this study, glaucine was found only in the TAE of *G. corniculatum* var. *corniculatum*, and not at high amount (∼1%) in comparison with *G. flavum* species which is one of the most analysed *Glaucium* species and its major alkaloid glaucine which has an aporphine ring, investigated for many bioactivities, and glaucine, was found to be as an antitussive agent, and its drugs were commonly used by people (Meyer et al. 2013). However, most of the extracts of the *Glaucium* species were investigated rather than their isolated alkaloids. Glaucine and other aporphine alkaloids were studied for cytotoxic/anticancer, anti-inflammatory, antinociceptive, antiplasmodial and anti-AChE activities. A number of protopine alkaloids were investigated for anti-inflammatory, cardiovasoprotective, antithrombotic, neuroprotective, antimicrobial and antispasmodic activities (Akaberi et al. 2021).

Recent studies also indicate that *Glaucium* species may be used for the treatment of neurodegenerative diseases, such as Alzheimer’s and/or Parkinson’s disease, due to their bioactive alkaloids (Tuzimski et al. 2022). Some studies with the *G. grandiflorum* subsp. *refractum* var. *grandiflorum* extracts exhibited high antioxidant, anti-AChE , anti-inflammatory and DNA-damaging properties (Aslan 2012; Orhan et al. 2004; Ozsoy et al. 2018).

In our study, the extracts of both *Glaucium* species (*G. corniculatum* var. *corniculatum* and *G. grandiflorum* subsp. *refractum* var. *torquatum* (Aslan 2012)) also showed high antioxidant activity (Figures 4 and 5) as well as high anti-AChE activity (Figures 7 and 8). Therefore, a correlation was observed between these two activities.

In the treatment of Alzheimer’s disease, AChE inhibition is the most effective way for patients because AChE inhibitory property has been accepted as an important marker for detecting active substances in neurodegenerative diseases (Anand et al. 2014). In previous studies, it has been proven that when exposing various cell types to oxidative stress, AChE expression increases in both gene and protein levels (Kocanci et al. 2022). In this study, glaucine and glauciumoline showed significant AChE inhibitory activity (Figure 9) while the other isolated alkaloids showed weak or no activity.

## Conclusions

Oxidative stress is a significant property in the mechanism of neurodegenerative diseases. Therefore, the interest in medicinal plants, particularly their alkaloids (such as galantamine), has increased in finding more active and safe new agents in the treatment of neurodegenerative disorders/diseases. In one of the recent studies, alkaloid extracts of *G. corniculatum* showed suppressed oxidative stress-induced neuronal apoptosis (Dolanbay et al. 2021). In another recent review article, neuroprotective and anti-cholinesterase activities, besides some other activities of *Glaucium* species, were reported (Akaberi et al. 2021).

In this study, we obtained a new alkaloid (**3)**, trivially named glauciumoline, having a protopine-type isoquinoline structure, and its novelty comes from the formation of a 10-member ring by B and C rings with an epoxy group between C-13 and C-14. Although most of the isolated alkaloids have not shown even weak anticholinesterase activity results, glaucimoline and glaucine exhibited relatively stronger AChE inhibitory activity results.

Obtained activity results of the extracts and isolated compounds from two *Glaucium* species have been compared with the previous studies on *Glaucium* species. The two extracts (TAE and QAE) prepared from both *Glaucium* species showed higher anticholinesterase activity than the pure alkaloids (Figures 9 and 10). The tertiary and quaternary amine extracts of both species shown strong AChE activity (13.83 ± 0.02–14.87 ± 0.03) which are comparable with the reference compound galantamine. However, only TAE of *G. corniculatum* var. *corniculatum* showed very strong butrylcholinesterase activity.

Both species extracts also exhibited high antioxidant capacity in general. In particular, they showed high lipid peroxidation inhibitory activity by a β-carotene-linoleic acid test method (Figure 5) as well as high CUPRAC and ABTS antioxidant capacity (Figure 4). However, isolated alkaloids have not shown CUPRAC and ABTS activity. In contrast, they showed good lipid peroxidation inhibitory activity (Figure 6).

As a result, both *Glaucium* extracts showed higher antioxidant and anticholinesterase activity than their pure isolated alkaloids, indicating that TAEs of both *Glaucium* species probably contain other secondary metabolites besides alkaloids. Thus, the strong activity of the extracts might be observed due to the total synergistic effect of all of these compounds.
